# Convolution-Based Encoding of Depth Images for Transfer Learning in RGB-D Scene Classification

**DOI:** 10.3390/s21237950

**Published:** 2021-11-28

**Authors:** Radhakrishnan Gopalapillai, Deepa Gupta, Mohammed Zakariah, Yousef Ajami Alotaibi

**Affiliations:** 1 Department of Computer Science & Engineering, Amrita School of Engineering, Amrita Vishwa Vidyapeetham, Bengaluru 560035, India; g_radhakrishnan@blr.amrita.edu; 2 Department of Computer Engineering, College of Computer and Information Sciences, King Saud University, P.O. Box 57168, Riyadh 11543, Saudi Arabia; mzakariah@ksu.edu.sa (M.Z.); yaalotaibi@ksu.edu.sa (Y.A.A.)

**Keywords:** depth encoding, multimodal learning, RGB-D images, scene classification, transfer learning

## Abstract

Classification of indoor environments is a challenging problem. The availability of low-cost depth sensors has opened up a new research area of using depth information in addition to color image (RGB) data for scene understanding. Transfer learning of deep convolutional networks with pairs of RGB and depth (RGB-D) images has to deal with integrating these two modalities. Single-channel depth images are often converted to three-channel images by extracting horizontal disparity, height above ground, and the angle of the pixel’s local surface normal (HHA) to apply transfer learning using networks trained on the Places365 dataset. The high computational cost of HHA encoding can be a major disadvantage for the real-time prediction of scenes, although this may be less important during the training phase. We propose a new, computationally efficient encoding method that can be integrated with any convolutional neural network. We show that our encoding approach performs equally well or better in a multimodal transfer learning setup for scene classification. Our encoding is implemented in a customized and pretrained VGG16 Net. We address the class imbalance problem seen in the image dataset using a method based on the synthetic minority oversampling technique (SMOTE) at the feature level. With appropriate image augmentation and fine-tuning, our network achieves scene classification accuracy comparable to that of other state-of-the-art architectures.

## 1. Introduction

Autonomous mobile robots are increasingly used in many real-world applications. They are used in different sectors including hospitals, restaurants, homes, agriculture, defense, etc. Technological advances have enabled these robots to carry out complex tasks such as search and rescue, surveillance, and transporting heavy items in warehouses. Since such robots are capable of navigating without any human intervention, they are increasingly deployed to assist people in performing mundane repeated jobs [1]. Their capability of autonomy comes from their ability to memorize and identify work environments. A domestic robot employed to take care of elderly persons navigates in indoor scenarios and recognizes objects present in rooms [2,3].

Autonomous mobile systems aided by multimodal sensing capabilities must understand the environment accurately to perform their tasks efficiently [4]. Understanding scenes from a 3D perspective adds to the capabilities of autonomous agents in domains such as autonomous vehicles, service robots in domestic, and industrial environments and health care systems. However, 3D scene understanding is a challenging task due to factors such as scale variations across different scenes, cluttering in the environment, and occlusions. Song et al. [5] identified six distinct tasks that are often performed as part of overall scene understanding when dealing with images of indoor scenes. These tasks are scene categorization, semantic segmentation, object detection, object orientation, room layout estimation, and total scene understanding. The present paper focuses on scene categorization or scene classification. Scene classification focuses on classifying an entire image into one of the predefined categories as opposed to classifying patches of images. Scene classification is necessary in many applications where autonomous agents are deployed. For example, consider a service robot moving in an unfamiliar indoor environment such as a large building consisting of different types of rooms. The robot should be able to identify the type of room that it is currently in, including an office room, cafeteria, bedroom, classroom, etc.

Classification of rooms is a challenging problem, as there are significant variations in layouts and objects present in each type of room [6]. Classification of scenes can be accomplished using (a) high-level features of the scenes, such as detected objects [7], (b) global image features, or (c) local image features. Classical scene categorization systems extract image features and then use them as input to a classifier including support vector machines (SVM), random forest, etc., for classification. The success of these systems depends on the right choice of features relevant to the task. As opposed to classical image/scene recognition systems, neural networks learn features automatically. Deep neural networks with millions of parameters require very large datasets for the accurate learning of features. With the availability of large datasets with millions of images, convolutional networks are able to learn features relevant to the task at hand with high discriminative capability. The successful use of deep convolutional neural networks (CNN) in image classification has resulted in neural networks being widely used for scene understanding tasks. Although scene understanding using RGB images has been able to achieve good classification accuracy, adding depth information to color can provide valuable additional details and thus can help better understand scenes. The availability of low-cost depth sensors has opened up a new research area of using depth information in addition to color (RGB) information for scene understanding. One of the problems faced by this research community is the lack of availability of large-scale datasets that contain RGB and depth (RGB-D) information. Even now, the sizes of RGB-D datasets are an order of magnitude smaller than those of color image datasets.

This study investigates CNN architectures to categorize scenes with dual-modality images, i.e., RGB and depth images. The SUN RGB-D dataset from Princeton University [5] is used as the benchmark dataset for training and validating the CNN models created. Although the SUN RGB-D dataset is one of the larger publicly available datasets for dual-modality images, the number of images available in this dataset is small compared with those available in RGB image datasets. When dealing with smaller datasets, image augmentation is often used. However, augmenting dual-modality image pairs that are consistent with each other needs special attention. Another challenge in dealing with RGB-D images is to select a suitable method to integrate the two modalities. The depth modality is typically stored as single-channel images, whereas RGB images have three channels. Most researchers convert single-channel depth images to three-channel images by extracting horizontal disparity, height above ground, and the angle of the pixel’s local surface normal (HHA). The high computational time complexity of HHA encoding is a major disadvantage for the real-time prediction of scenes; although, this may be a less important issue during the training phase. The current work proposes a new encoding to transform single-channel depth images into three channels. We also address the issue of class imbalance seen in the SUN RGB-D dataset. This class imbalance affects the classification accuracy of minority classes. We address this problem by extracting features from the dense layer of the network and then oversampling the feature dataset using minority classes using the SMOTE technique [8]. 

Our major contribution is the following: We propose a new encoding method for depth images that can be integrated with any convolutional neural network. We show that effective transfer learning using depth images combined with RGB images is possible with this encoding method. While the standalone performance of our approach is inferior to that of HHA encoding when a network is trained only with depth images, our approach performs equally well or better in a multimodal learning setup.

Additionally, we employ a data augmentation scheme in a dual-modality setup that ensures consistency between RGB and depth image transformations. The class imbalance problem seen in the SUN RGB-D image dataset is addressed by applying the SMOTE technique to the features extracted after training a deep convolutional network and then using these features to retrain an ablated network. To the best of our knowledge, ours is the first attempt to address the class imbalance in the SUN RDB-D dataset

The remainder of this paper is organized as follows. A discussion on related work is given in Section 2. Key features of the benchmark SUN RGB-D dataset are given in Section 3. The proposed architecture with a data augmentation scheme and methods for depth encoding and SMOTE oversampling are discussed in Section 4. Section 5 discusses the experimental setup. The scene classification performance with the new methods using the benchmark dataset is analyzed in Section 6. The conclusion is presented in Section 7.

## 2. Related Work

We review the work done in four areas related to scene classification: scene classification using features extracted, scene classification using neural networks, scene recognition using RGB-D images, and class balancing.

### 2.1. Scene Classification Using Features Extracted

Most autonomous systems use a variety of sensors to perceive the environment [9,10]. Learning about the environment can be done using data captured with very simple ultrasonic sensors [11], cameras, or even sophisticated lidar systems such as those used in self-driving cars. In many situations, data coming from these multimodal sensing devices are processed as large data streams for the clustering and classification of scenes [12,13]. In the early years of visual scene understanding, researchers mainly used features extracted from images for scene recognition tasks. These features are broadly divided into two categories [14]: local feature descriptors that represent relevant parts of the image and global descriptors that represent the whole image. Local feature descriptors have been found to be very effective in many tasks such as object recognition. The scale invariant feature transform (SIFT) method generates local feature descriptors that are used in many object recognition systems [15]. SIFT features are generally invariant to rotation, scaling, and translation of images and are partially invariant to illumination changes. Other popular local descriptors include spin-images [16], histograms of oriented gradients (HOGs) [17], and speeded up robust features (SURFs) [18]. Popular global attribute descriptors include GIST [19] and CENsus TRansform hISTogram (CENTRIST) [20]. The performance of global attribute descriptors is often constrained by complex visual constitutions of images. In the case of scene categorization tasks, feature descriptors derived from scene images are given to a classification algorithm to predict the category of the scene. Scene categorization performance depends on the quality of the feature descriptors extracted. Xie et al. [21] groups scene recognition algorithms into six categories based on the features extracted: patch features, spatial layout patterns, discriminative regions, object correlations, global attributes, and hybrid deep models.

### 2.2. Scene Classification Using Neural Networks

Shallow neural networks have been used for classification in many domains including image classification [22]. The main attraction of neural networks is that the features are not hand-picked by the user, but automatically learned by the system. Deep neural networks are used in diverse application areas such as image enhancement and image classification involving single label and multi-label [23,24]. The current trend in scene understanding is to use deep neural networks. Deep neural networks used in visual place recognition typically have a set of convolutional layers near the input layer followed by a small number of fully connected layers near the output layer. Each convolutional layer at the bottom end of the network learns local features at increasing levels of abstraction, whereas the top layers learn more global features. Most deep learning networks have millions of parameters to learn, and the learning phase requires a large number of images. Popular deep learning networks such as AlexNet [25], VGGNet [26], InceptionNets [27], and ResNet [28] have achieved significant results in image classification with millions of images used for training the network. Although these networks were initially developed for image classification, they are also used for scene classification with the availability of a new dataset for scenes or places. Places365 is a benchmark dataset that contains approximately 2.15 million images belonging to 365 different scene classes [29]. Pretrained models of the VGG16 network, GoogLeNet, and AlexNet trained with the Places365 dataset are available. A standard practice is to use one of the pretrained CNNs to extract features and then to use these features as input for classifiers such as random forests and support vector machines [30,31].

### 2.3. Scene Recognition Using RGB-D Images

While image classification with RGB images has achieved excellent results, researchers are exploring the benefit of adding depth information for overall scene understanding. Since depth cameras such as Kinect are less impacted by low illumination, the use of depth images along with RGB images can provide better features in scenes where illumination is not sufficient. Since capturing depth images requires special devices, the sizes of datasets that include depth information have not yet reached the scale of RGB image datasets. Silberman et al. [32] introduced one of the first public RGB-D datasets, the NYU Depth V2. This dataset contains 1449 RGB-D images from indoor scenes. The authors used their dataset to show how appearance cues, room-aligned 3D cues, surface fitting, and scene priors can be used to parse complex environments. In 2014, Song et al. [5] created a much larger dataset for indoor environments that includes the NYU Depth V2 as a subset. The authors used their SUN RGB-D dataset for scene categorization, semantic segmentation, object detection, object orientation, room layout estimation, and total scene understanding. They used handcrafted features as well as a CNN for classification. Their approach also used a hybrid approach—features are extracted from the CNN and then used for classification with SVM.

When dual modalities are used, there are three possible approaches to design a network architecture: (1) The depth channel is concatenated with RGB channels to form a single input of four channels. This approach is often called the early fusion approach [33,34]. (2) Late fusion is performed, in which two independent networks are trained and the features obtained from them are concatenated before final classification. (3) Separate bottom layers are used for two modalities, and then, these two paths are merged to form common top layers. Gupta et al. [35] proposed a new encoding scheme to convert single-channel depth images to three-channel images. Their encoding, known as HHA encoding, created three-channel images consisting of horizontal disparity, height above ground, and the angle that the pixel’s local surface normal makes with the inferred gravity direction.

The state of the art in RGB-D scene classification incorporates a pretrained Places365 network at some stage. Zhu et al. [36] used a pretrained AlexNet trained on the Places205 dataset [29], which is a subset of the Places365 dataset, to classify scenes from the SUN RGB-D dataset. The Places205 dataset contains 205 scene categories, whereas the Places365 dataset contains 365 scene categories. The pretrained AlexNet has five convolutional layers followed by three fully connected layers. The pretrained network is modified by removing the last fully connected layer and extracting features from the second fully connected layer. The depth images are encoded with HHA encoding consisting of three channels so that the pretrained AlexNet can also be used for depth images. Separate CNNs for RGB images and depth images are used, and features are extracted. The features obtained from the final dense layer of the CNNs for two modalities, i.e., RGB and depth, are fused to obtain the final set of features that are passed to an SVM classifier to perform scene classification.

Humans utilize object knowledge for scene understanding. Inspired by this fact, Liao et al. [37] incorporated object-level information. The authors constructed their network with regularization of semantic segmentation. A combination of depth images and normal vector images is used to encode the depth data. One of the drawbacks of object-based scene understanding is that two different classes of scenes can have similar types of objects but different layouts. Classroom and lecture theatre are examples from the SUN RGB-D dataset that have similar object content. Li et al. [38] proposed a discriminative feature-learning network to learn distinctive features and correlated features of color and depth modalities in two stages. Discriminative features for the modalities are learned in the first phase. Independent and correlative feature information is extracted in the second phase. A more recent work by Song et al. [39] studied the effect of fine-tuning a pretrained AlexNet at multiple setups for the classification of RGB-D images. Their experiments focused on three different configurations for fine-tuning: (a) FT-top, where only selected layers near the output are fine-tuned; (b) FT-bottom, where a few convolutional layers near the input are fine-tuned; and (c) FT-shallow, where a few convolutional layers are kept and fine-tuned while the others are removed. The authors observed that fine-tuning bottom layers is equally important to training top layers. It was observed that the majority of the filters in convolutional layer 1 extract low-level patterns in RGB scenes reasonably well. When only the top layers of the depth network are fine-tuned with a relatively small number of depth images, weight adaptation at the bottom layers was been found to be slow. The authors also proposed an architecture to learn depth features using weak supervision via patches and a two-step training approach. Working on patches of images helps to increase the training data size and reduce the number of parameters in the network. The parameters learned using patches were later transferred to another network that can take full-size images for further fine-tuning. Xiong et al. [40] proposed a framework to learn local and global features simultaneously. They introduced a new module in the CNN to adaptively select key local features. The key local feature selection module was trained using spatial attention similarity loss. Xiong et al. [41] subsequently proposed a differentiable local feature selection (DLFS) module to adaptively select important local object-level and theme-level features. Most of the approaches proposed for scene understanding from RGB-D images are extensions of the methods used for RGB images using an appropriate fusion strategy [42]. Du et al. [43] proposed a framework that integrates modality-specific recognition and cross-modal translation. Ayub and Wagnar [44] first formed clusters of different scene categories and then their centroids are calculated. Classification of test images was been done by choosing the closest centroid. A graph neural network-based approach was proposed in [45]. Naseer et al. [46] noted in their survey on indoor scene understanding in 2.5D that when the domain on which the training was done was not closely related to the target domain, optimally adapting a pretrained model is a challenging task.

### 2.4. Class Balancing

Training a convolutional network with a few thousand images either from scratch or using transfer learning with a pretrained network that has been trained with a different modality has limitations. An additional issue is the problem of class imbalance, where some of the classes have significantly fewer samples than other classes. A standard technique used to increase the size of a dataset to obtain better regularization is image augmentation. Class imbalance is often addressed by an oversampling method such as SMOTE [8]. Oversampling is the most commonly used class balancing technique employed by the deep learning community [47]. Other techniques such as using new loss functions [48] and multiscale feature fusion [49] have also been proposed. Wong et al. [50] investigated and compared the benefit of data augmentation in data space and feature space. They observed that an appropriate data transform method to augment data in the data space often provides greater benefit than applying oversampling or undersampling in the feature space.

Most of the recent work for scene classification with RGB-D images discussed in previous paragraphs employ transfer learning using Places-CNN and fine-tuning them for dual-modality. HHA encoding of depth images required for transfer learning is computationally intensive. Our proposed encoding method is computationally efficient, as the convolutional layer used for encoding has fewer than 100 parameters compared to millions of parameters learned in the rest of the network. The limited sizes of the datasets used for training pose challenges in obtaining good classification performance. While many researchers have applied either data augmentation or oversampling, very few studies have been done combining both methods. We employ a data augmentation method that maintains consistency between pairs of images from the two modalities at the image level and SMOTE oversampling at the feature level.

## 3. Benchmark Dataset

The SUN RGB-D dataset from Princeton University (https://rgbd.cs.princeton.edu/data/SUNRGBD.zip) is used in this study. This dataset was created using four different devices: an Intel RealSense 3D camera for tablets, an Asus Xtion Live Pro for laptops, and Microsoft Kinect versions 1 and 2 for desktop. In total, there are 10,335 RGB-D images. This dataset includes images from NYU Depth V2 [32], Berkeley B3DO Dataset [51], and SUN3D videos [52]. These images are scenes from universities, houses, and furniture stores in North America and Asia. Although the dataset contains images from 45 different scenes, only 19 scene classes are well represented. Each of these 19 classes has more than 160 images, whereas the remaining 26 classes have a smaller number of images. These 19 classes are shown in Table 1.

The number of instances in this subset of 19 classes ranges from 169 images of type *home_office* to 1084 images of type *bedroom*. To compare with previous works [38,39,40,41], we also used these 19 classes and a train/test split of 4845 images for training and 4659 images for testing. We refer to this subset of 9504 images as the SUN RGB-D benchmark dataset. Since the SUN RGB-D dataset includes all the 1449 images from the NYU Depth V2 dataset, separate evaluation of the proposed method has not been carried out using NYU Depth V2 dataset.

## 4. Architecture of the Proposed Method

**Figure 1:**
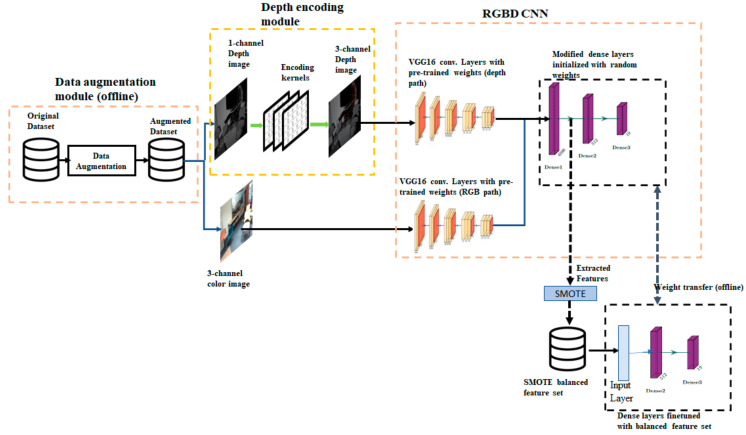
The architecture of the proposed method: The architecture comprises of four components (i) an offline data augmentation module, (ii) a depth encoding module implemented as a layer in the convolutional neural network, (iii) backbone convolutional neural network (referred to as RGBD CNN in the paper) based on VGG16 Net, and (iv) a class balancing module.

The state-of-the-art RGB-D scene classification uses a standard deep convolution network such as Alexnet, VGGNet pretrained with Places dataset as the backbone network. We use a VGG 16-layer network pre-trained on the Places365 dataset. Our proposed architecture integrates a module for data augmentation to compensate for the limited number of images available in RGB-D scene training datasets. The key component of the proposed architecture is an additional convolution layer added to the backbone network that takes a single channel depth image and converts it to a three-channel image. The overall architecture of the proposed method is shown in Figure 1. The architecture has four distinct components: (1) VGG16 convolutional neural network, (2) data augmentation module, (3) depth encoding module, and (4) offline module to adjust class imbalance at the feature level and fine-tune dense layers. Each of these components is described here.

### 4.1. VGG Convolutional Network

We use a VGG 16-layer network pre-trained on the Places365 dataset (referred to as VGG16-PlacesNet) as the baseline architecture for transfer learning. Since VGG16-PlacesNet uses three-channel images as its input, depth images encoded with three channels are to be used. When RGB and depth images are involved, the difference in the number of channels used to represent the image needs to be considered. RGB images use three channels, whereas depth images are usually stored with a single channel. Due to these differences, a network that is used for color modality cannot be used for depth modality as it is.

#### Network with Two Convolutional Paths

A commonly used approach with dual modalities is to use separate bottom layers for two modalities and then merge these two paths to form common top layers. Outputs of convolutional layers of both paths are concatenated together and fed to common fully connected layers. We refer to this network as RGBD CNN. As shown in Figure 1, the RGBD CNN has separate convolutional paths for the RGB and depth modalities. The convolutional layers are the same as in VGG16-PlacesNet, and dense layers have been modified to meet the requirements of the SUN RGB-D dataset. The output layer is modified to have 19 neurons to match the number of classes in the dataset. An ablation study was carried out to modify the pooling layers. Details of the ablation study and modifications are discussed in the experimental setup section.

### 4.2. Data Augmentation Module

The number of images present in SUN RGB-D dataset is relatively small for deep neural networks. Augmenting the training set by adding synthetic scenes is one of the methods used in such situations. Another approach is to use image augmentation methods to transform the original images to generate additional images which are similar to the old ones. Although *Keras* and *TensorFlow* provide image augmentation methods with their image generator class, we have not used them in this study, as the input consists of pairs of images belonging to two modalities that should undergo exactly similar transformations. Instead, data augmentation methods are used on the images in the training partition of the dataset to create a new larger static training dataset. A limited set of data augmentation methods that are considered to be safe for the task of scene recognition were used. Data augmentation was performed in an offline fashion prior to the training process.

#### Data Augmentation Method

**Figure 2:**
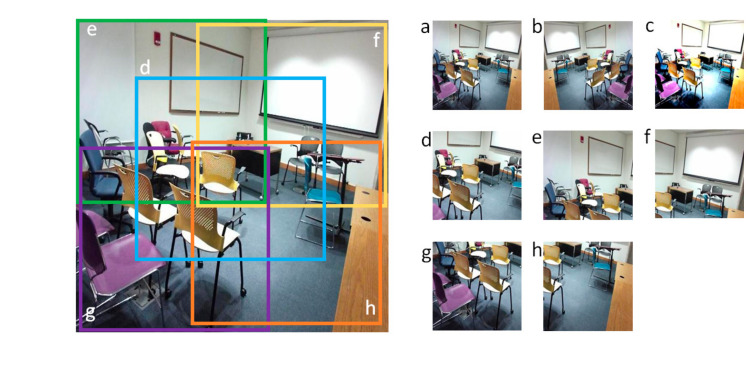
Images created using data augmentation (**a**) original image; (**b**) horizontal flip; (**c**) contrast and brightness change; (**d**) zoom to the middle; (**e**) cropped top left; (**f**) cropped top right; (**g**) cropped bottom right; (**h**) cropped bottom left.

Each RGB and depth image is subjected to seven different augmentation methods such that the application of each method creates a new image and is independent of the other methods used. Hence, the enhanced training dataset is eight times larger than the original dataset. The set of eight images in the new training dataset corresponding to a single image in the unprocessed dataset are as follows: (a) original image, (b) horizontal flip, (c) contrast and brightness change, (d) zoom to the middle to obtain 60% of the image area, (e) crop 60% of the image from the top left, (f) crop 60% of the image from the top right, (g) crop 60% of the image from the bottom right, and (h) crop 60% of the image from the bottom left. A set of new images is shown along with the original image in Figure 2. The large image on the left is the original image. Boxes marked in the image show different crop areas used to create a new image set. Since contrast and brightness changes are not appropriate for depth images, new RGB images created with the contrast change operation are paired with original depth images.

During the training process, a pair of RGB images and depth images from an instance exactly similar to an image transformation are taken together from the augmented dataset. Depth images are given as input to the depth encoding module at the time of training and evaluation, and the resultant three-layer encoded images are given as input to the first convolutional block of the RGBD CNN depth path.

### 4.3. Depth Encoding Module

VGG16-PlacesNet takes three-channel inputs. Depth images have to be converted to three channels to employ transfer learning using pretrained weights for the depth modality. The standard practice is to use HHA encoding to make depth images compatible with RGB images. However, the HHA encoding algorithm has high computational complexity and can run into performance issues when real-time scene evaluation is needed. In the case of HHA encoding, preprocessing of the depth image is performed before the input is given to the neural network.

**Figure 3:**
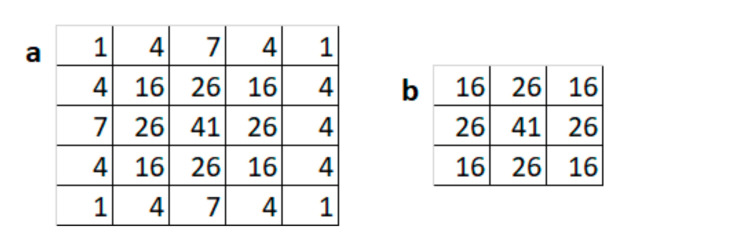
Preprocessing kernels: (**a**) 5 × 5 Gaussian smoothening kernel; (**b**) 3 × 3 Gaussian kernel.

The straightforward method to generate three channels is to create new channels by replicating the contents of the original depth channel values. This method is suboptimal and the two new channels do not provide any additional information. We propose a convolution-based encoding (CBE) method in which single-channel depth images are converted to three-channel images using three kernel filters: (1) Channel 1 of the modified image takes exactly the same pixel values as the normalized pixel values of depth images; (2) Channel 2 takes pixel values after applying a 5 × 5 Gaussian smoothening filter, as shown in Figure 3a; and (3) Channel 3 has pixel values after applying a 3 × 3 Gaussian smoothening filter, as shown in Figure 3b. The gaussian smoothing applied with two filters of different sizes enhances the image structures in the new channels at two different scales. Our experiments confirmed that the use of gaussian filters performs better compared to replicating the single-channel depth data directly to the three input channels. The main advantage of the proposed encoding over HHA is its computational efficiency.

#### Implementing Encoding Filters Using a Convolutional Layer

**Figure 4:**
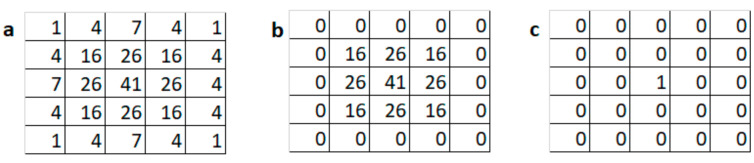
Weights of encoding layer filters: (**a**) 5 × 5 Gaussian smoothening filter; (**b**) 3 × 3 Gaussian filter with zero-padding; (**c**) Identity filter.

**Figure 5:**
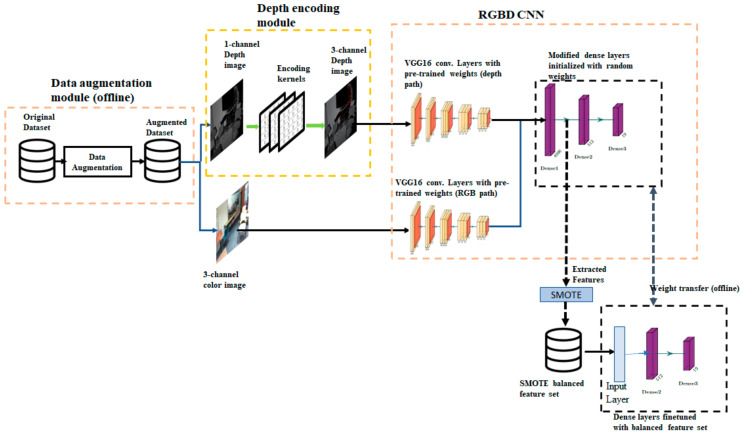
RGBD CNN modified with an added convolutional layer for CBE.

An advantage of this encoding method is that the preprocessing filters can be easily incorporated as part of the convolutional neural network. This can be achieved by adding a new convolutional layer to the depth path of the RGBD CNN. Since all the filters in the convolutional layer have to have the same size, the 3 × 3 Gaussian smoothening filter is converted to a 5 × 5 filter by adding zero-padded rows and columns. The 5 × 5 Gaussian filter and the 3 × 3 Gaussian filter with zero-padding are shown in Figure 4a and 4b respectively. Similarly, the 5 × 5 identity filter shown in Figure 4c is used as the pass-through filter. The weights of the preprocessing convolutional layer were preloaded. The RGBD CNN enhanced with the addition of a new convolutional layer is shown in Figure 5. In essence, this convolutional layer implements the depth encoding module shown in Figure 1. Single-channel depth images are the input to the depth path of the modified RGBD CNN. 

Incorporating a preprocessing convolutional layer to perform depth encoding has many benefits: (a) The weights of the convolutional layer can be made trainable. This enables the encoding filters to adapt to the weights of the pretrained network used for transfer learning. (b) The encoding method is computationally efficient, and (c) The need for offline processing is eliminated.

### 4.4. SMOTE Oversampling and Fine-Tuning of Dense Layers

The number of samples available for each scene category in the SUN RGB-D dataset have significant variations, which impact the classification accuracy for minority classes. For example, scene types *home_office* and *lecture_theatre* have fewer than 180 images, whereas *bedroom* and *office* have more than 1000 images. Oversampling is a popular method to address class imbalance. This study uses the SMOTE method for oversampling. Since the SMOTE method is not suitable at the image level, we used it at the feature level by extracting the output of the first dense layer in the trained network. This layer has 4096 neurons, and hence the feature vector has 4096 features. The process for SMOTE oversampling and fine-tuning of dense layers is as follows:

Step 1. Train RGBD CNN using the augmented training set.
Step 2. Using the trained network, extract a feature vector for each sample in the training set to create a feature dataset.
Step 3. Apply SMOTE oversampling on the feature dataset to create a balanced feature set.
Step 4. Create a new neural network consisting of only dense layers matching the dense layers of RGBD CNN. Copy the weights from the trained RGBD CNN to the new network.
Step 5. Train the newly created network using the balance feature set.
Step 6. Copy the weights from the new network to the dense layers of the trained RGBD CNN.

## 5. Experimental Setup

The proposed convolutional neural network is implemented using *TensorFlow* with *Keras*. The training was carried out using a desktop grade Nvidia graphics processing unit. During the training of the network, we used a batch size of 32 and an initial learning rate of 0.0005 with a decay rate of 95% after every five epochs. The optimizer used is adam optimizer. Regularization is done with a dropout of 50% after every dense layer and 30% dropout before the last convolutional layer. SMOTE oversampling was performed using an imbalanced-learn library [53], which is available as a *contrib* library in *scikit-learn* [54].

### 5.1. Dataset for Training and Validation

The proposed architecture was evaluated using the SUN RGB-D benchmark dataset. Experiments were performed with HHA encoding as well as convolution-based encoding (CBE). Since HHA encoding is performed offline, a new dataset (HHA_dataset) is created for experiments that use HHA encoding. HHA_dataset is a replica of the SUN RGB-D benchmark dataset obtained by substituting single-channel depth images with corresponding three-channel depth images obtained through HHA encoding. Both datasets were split into three sets with 4335 images in the training set, 510 images in the validation set, and 4659 images in the test set. Data augmentation of the training set was performed prior to the training process. The augmented training set has 34,680 RGB and depth image pairs.

### 5.2. Ablation Study on VGG16-PlacesNet Configurations for Transfer Learning

**Figure 6:**
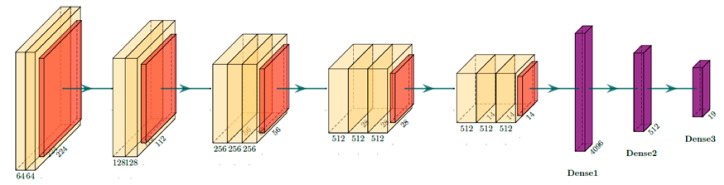
Single modality network based on VGG16-PlacesNet used for the ablation study.

One of the key points in transfer learning is to determine how the learned weights should be used. A study was carried out to decide on the best strategy to transfer weights from the pretrained VGG16-PlacesNet. Experiments were performed with single modality using a network architecture matching the VGG16-PlacesNet. VGG16-PlacesNet with a modified softmax layer used for this study is shown in Figure 6. The weights of some of the layers could be used without any modification or could be fine-tuned or discarded altogether. As the number of neurons in the output layer is different from the standard VGG16-PlacesNet, the weights of the output layer cannot be initialized with those from pretrained VGG16-PlacesNet. The weights of the convolutional layers were always preloaded from the pretrained VGG16-PlacesNet. The weights of the dense layers were either initialized with pretrained weights or with random values. The effect of freezing the preloaded weights of various convolutional layers by making some of the convolutional layers nontrainable was also studied. The best result was obtained when all the layers of the network were made trainable and the dense layers were initialized with random weights. Accordingly, all experiments with RGBD CNN were carried out with all layers trainable and randomly initialized weights for the dense layers.

### 5.3. Implementation of the Depth Encoding Module

**Figure 7:**
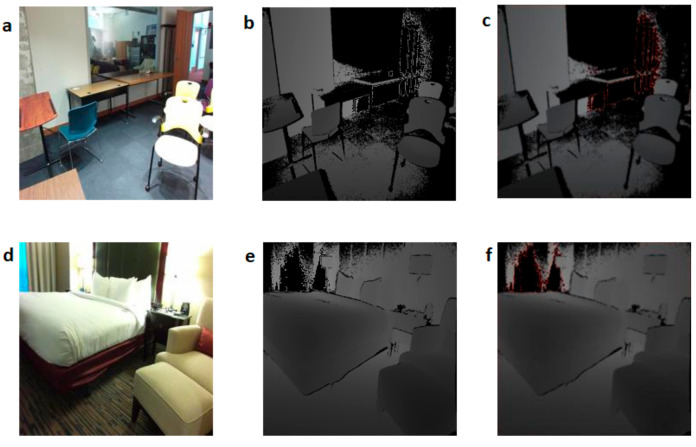
Images of a classroom and bedroom with and without new depth encoding: (**a**) RGB image of a classroom; (**b**) depth image of the classroom; (**c**) encoded depth image of the classroom; (**d**) RGB image of a bedroom; (**e**) depth image of the bedroom; (**f**) encoded depth image of the bedroom.

Experiments with convolution-based encoding were performed with an RGBD CNN with a CBE layer. Two sample images from the benchmark dataset and the corresponding images encoded with convolution-based encoding are shown in Figure 7. The encoded images were extracted as the output of the CBE layer. Figure 7a shows an RGB image of a classroom, and Figure 7b shows the corresponding depth image. The single-channel depth image was processed using the proposed encoding scheme to obtain a three-channel depth image shown in Figure 7c. Figure 7d–f shows the RGB image, single-channel depth image, and the encoded three-channel depth image of a bedroom. The encoded depth images preserve most of the visual features seen in the corresponding RGB images.

## 6. Experimental Results and Analysis

Training and testing were performed with transfer learning in different setups. These setups are as follows:

1. RGBD CNN with HHA: This set of experiments used the benchmark dataset without data augmentation. Depth images were encoded using HHA encoding. Hence the CBE encoding module was not used and the three channel HHA encoded images are given as input to the first layer of RGBD CNN
2. RGBD CNN with HHA + DA: This set of experiments used a setup similar to the one in setup 1. However, the training dataset with data augmentation was used for training.
3. RGBD CNN with CBE: RGBD CNN with added CBE layer was used in this setup. Dataset without data augmentation was used for training.
4. RGBD CNN with CBE + DA: Network architecture in this setup is similar to the one in setup 3, i.e., RGBD CNN with added CBE layer. Dataset with data augmentation was used for training
5. RGBD CNN with CBE + DA + SMOTE: This setup used RGBD CNN with added CBE layer and data augmentation and class balancing using SMOTE.

### 6.1. Experimental Results with Data Augmentation and Convolution-Based Encoding

#### 6.1.1. Data Augmentation

Experiments were performed with and without data augmentation using HHA encoding as well as convolution-based encoding When the RGBD CNN was used without data augmentation and depth images converted with HHA encoding, the of scene classification accuracy obtained was 54.7%. RGBD CNN with HHA encoding and data augmentation gave a classification accuracy of 57.3%. The number of images in the training set without augmentation was 4335, whereas the augmented training dataset had 34,680 images. An eightfold increase in the size of the training increased classification accuracy by approximately 2.6%.

#### 6.1.2. Convolution-Based Encoding

Experiments were conducted with RGBD CNN with a CBE layer with and without data augmentation. A comparison of the classification results of CBE with HHA encoding without data augmentation shows that CBE (with an accuracy of 55.07%) performed marginally better than that with HHA encoding (with an accuracy of 54.7%). It appears that the depth features highlighted by the new encoding scheme are complementary to the RGB features. CBE with data augmentation gave an accuracy of 58.53%, which is higher than that obtained with HHA encoding with data augmentation. Experiments were also performed in two configurations to understand the effect of using trainable filters for encoding. In the first configuration, the encoding layer was made nontrainable, whereas in the second configuration, even the encoding layer was made trainable. No significant difference in classification accuracies between these two configurations was observed.

A summary of the results obtained from these experiments is shown in Table 2.

**Figure 8:**
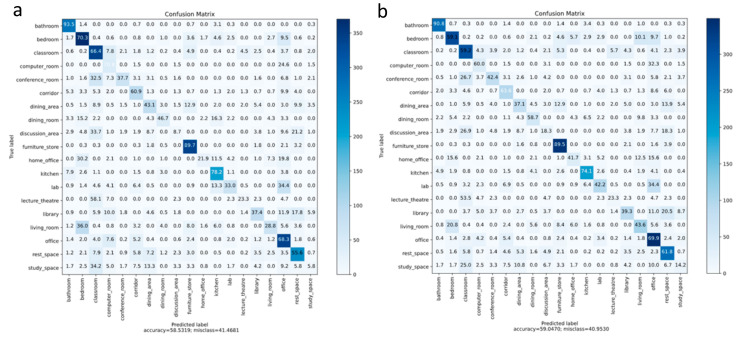
Confusion matrix with the SUN RGB-D dataset: (**a**) RGBD CNN with CBE and data augmentation; (**b**) RGBD CNN with CBE and data augmentation and class balancing with SMOTE.

The confusion matrix for the RGBD CNN with CBE and data augmentation is shown in Figure 8a. It is noted that the network performed well for certain classes such as *bathrooms*, which had a classification accuracy of 94.9%, whereas it did not perform well for other classes such as *discussion_area*, which had a classification accuracy of just 8.7%. Approximately 33.7% of scenes belonging to *discussion_area* were wrongly classified as a *classroom*. One of the reasons for this result is that classrooms are similar to discussion areas in appearance. It can be observed that the classes with a smaller number of images in the training data performed poorly due to the class imbalance. The training became biased toward scenes with a larger number of images in the training dataset.

### 6.2. Experimental Results with Oversampling

**Figure 9:**
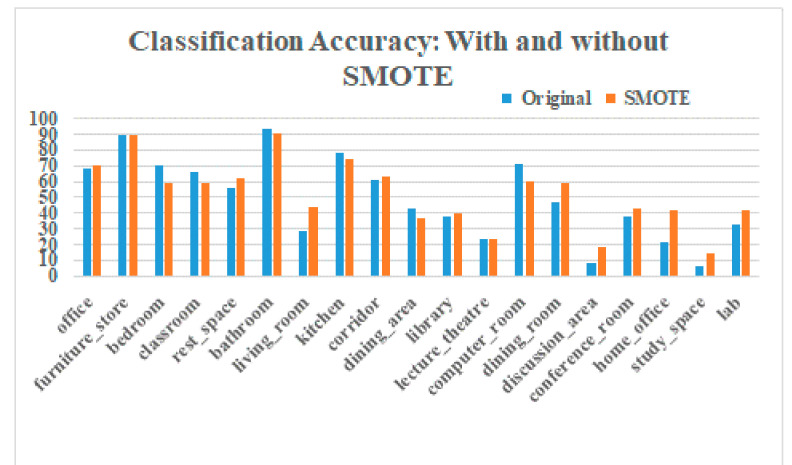
Classification accuracy of individual classes with and without SMOTE balancing.

SMOTE oversampling was applied on features extracted at the output of the first dense layer of the trained RGBD CNN. The features extracted with 34,680 images from the augmented training data were stored in an array of size 34,680 × 4096. The minimum number of instances for a class in the augmented training set was 296, and the maximum was 4152. We experimented with various oversampling ratios and finally chose a ratio where the classes with 500 or fewer samples were upsampled to 1000 samples and classes with 1500 or fewer samples were upsampled to 1500 samples using the SMOTE approach. The resulting feature matrix was of size 41,080 × 4096. The confusion matrix of the test results with the network retrained with this new feature set is shown in Figure 8b. The overall accuracy increased marginally to 59.05%, as shown in Table 2. However, the classification accuracies of minority classes showed good improvement. For example, the classification accuracy of *discussion_area* improved from 8.7% to 18.3% with SMOTE. The number of scenes from *discussion_area* wrongly classified as *classroom* decreased from 33.7% to 26.9%. A comparison of classification accuracies with and without SMOTE for each scene category is shown as a bar chart in Figure 9. Classes in the bar chart are shown in the order of decreasing class size.

### 6.3. Comparison with Existing Methods

The scene recognition accuracies obtained by recent works on the SUN RGB-D benchmark dataset are summarized in Table 3. Our proposed method with CBE encoding and SMOTE oversampling achieves performance close to the best-in-class. The dual-modality RGBD CNN showed improved accuracy with a combination of data augmentation, convolution-based encoding and class balancing with SMOTE oversampling. Compared to the other recent works done in this area, we use a simpler dual-path network. Data augmentation and SMOTE oversampling are the key contributors to improving the accuracy. The correction in class balance helped to improve the classification accuracy of minority classes. The convolution-based encoding converts the single-channel depth images using three filters. The computation time required to apply three filters is insignificant compared to the time taken in hundreds of filters used in the subsequent layers of the network. The traditional approach of using HHA encoding would require the depth images to be first converted to three-channel images before feeding it to the network of scene classification. On a low-end desktop computer, encoding of each image took close to 100 milliseconds. The computation time taken by HHA encoding depends on the number of iterations used by the optimization algorithm and it varies from image to image. The convolution-based encoding makes it possible to classify newly acquired images in a few milliseconds, making our model attractive for real-time applications.

## 7. Conclusions

The availability of low-cost depth cameras has opened up the possibility of adding a depth dimension to RGB images for scene understanding. However, integrating the depth modality with color is a challenging task. Transfer learning from pretrained RGB networks requires depth images to be encoded with data of dimensions similar to those of RGB. Popular HHA encoding is computationally intensive and poses challenges in real-time scene classification. We proposed a new encoding method for converting single-channel depth images to three-channel images so that pretrained networks on RGB networks can be used for transfer learning for RGB-D classification. The new method is computationally efficient and can be plugged in as a convolutional layer in the convolutional neural network. We used a data augmentation technique at the data space level to partially address the limited quantity of training data. The class imbalance issue is addressed through SMOTE oversampling on the features extracted from the dense layer of the convolutional neural network. The scene classification accuracy obtained with the proposed method for RGB-D scene classification is comparable to that of the state of the art.

The main novelty of our method is the convolution-based encoding scheme for depth images, which is computationally efficient. Integration of convolution-based encoding with other state-of-the-art network architectures is yet to be explored. Although the proposed encoding is good for scene classification tasks with dual-modality images, its usefulness for other scene understanding tasks is yet to be evaluated.

## Figures and Tables

**Table 1:** Class labels and number of image instances in the SUN RGB-D benchmark dataset.

Sl. No.	Class Label	Numbers of Images
1	bathroom	624
2	bedroom	1084
3	classroom	1023
4	computer_room	179
5	conference_room	290
6	corridor	373
7	dining_area	397
8	dining_room	200
9	discussion_area	201
10	furniture_store	965
11	home_office	169
12	kitchen	498
13	lab	258
14	lecture_theatre	176
15	library	381
16	living_room	524
17	office	1046
18	rest_space	924
19	study_space	192

**Table 2:** Scene classification accuracies with different network configurations with and without augmented datasets.

Network Configuration	Classification Accuracy	
	Without DA	With DA
RGBD CNN with HHA	54.7%	57.3
RGBD CNN with CBE	55.07%	58.3
RGBD CNN with CBE + SMOTE		59.05%

DA—Data augmentation; CBE—Convolution-based encoding.

**Table 3:** Scene classification accuracies on SUN RGB-D with dual modality.

Method	Accuracy %
Zhu et al. [36]	41.5
Song et al. [39]	53.8
Li et al. [38]	54.6
Xiong et al. [40]	55.9
Xiong et al. [41]	57.3
Ayub et al. [44]	**59.5**
**Proposed method (RGBD CNN with CBE + DA + SMOTE)**	59.05
